# Long-term efficacy of HDM-SCIT in pediatric and adult patients with allergic rhinitis

**DOI:** 10.1186/s13223-023-00781-8

**Published:** 2023-03-11

**Authors:** Lei Ren, Chengshuo Wang, Lin Xi, Yunbo Gao, Yuan Zhang, Luo Zhang

**Affiliations:** 1 Department of Allergy, Beijing TongRen Hospital, Capital Medical University, Beijing, China; 2 Department of Otolaryngology Head and Neck Surgery, Beijing TongRen Hospital, Capital Medical University, Beijing, China; 3 Beijing Laboratory of Allergic Diseases and Beijing Key Laboratory of Nasal Diseases, Beijing Institute of Otolaryngology, No. 17, HouGou Hu Tong, Dong Cheng District, Beijing, 100005 People’s Republic of China; 4 Research Unit of Diagnosis and Treatment of Chronic Nasal Diseases, Chinese Academy of Medical Sciences, Beijing, China

**Keywords:** Allergic rhinitis, Efficacy, Long-term, Pediatric, Subcutaneous immunotherapy

## Abstract

**Background:**

Subcutaneous immunotherapy (SCIT) is a well-validated and effective disease modification treatment for house dust mites (HDM)-induced allergic rhinitis (AR). Long-term post-treatment comparisons in children and adults treated with SCIT have rarely been published. This study aimed to evaluate the long-term efficacy of HDM-SCIT administered under a cluster schedule in children compared to adults.

**Methods:**

This was an open-design, observational, long-term clinical follow-up study on children and adults with perennial AR treated with HDM-SCIT. The follow-up consisted of a three-year treatment duration plus a post-treatment follow-up of over three years.

**Results:**

Patients in the pediatric (n = 58) and adult (n = 103) groups completed a post-SCIT follow-up of over three years. The total nasal symptom score (TNSS), combined symptom medication score (CSMS), and rhinoconjunctivitis quality-of-life questionnaire (RQLQ) score decreased significantly at T1 (three-year SCIT completed) and T2 (follow-up completed) in the pediatric and adult groups. In both groups, the improvement rate of TNSS (T0-T1) was moderately correlated with the baseline TNSS (r = 0.681, p < 0.001 and r = 0.477, p < 0.001 for children and adults, respectively). Only in the pediatric group, TNSS was significantly lower at T2 compared with that right after SCIT cessation (T1) (p = 0.030).

**Conclusions:**

Children and adults with HDM-induced perennial AR could achieve a sustainable post-treatment efficacy for over three years (up to 13 years) following a three-year SCIT. Patients with relatively severe nasal symptoms at baseline may benefit more from SCIT. Children who have completed an adequate course of SCIT may gain further improvement in nasal symptoms after SCIT cessation.

## Background

Allergic rhinitis (AR) is a global health problem with a prevalence of up to 50% in some countries [1]. AR affects the quality of life of approximately 250 million (17.6%) people in China and is associated with a substantial economic burden to society [2]. The prevalence of AR among students (10–17 years old) is as high as 42.5% (self-report) in Central China [3]. House dust mites (HDMs) are the most common aeroallergens in patients with perennial AR. The positive rate of HDM sensitisation in children with AR is 93.1% in Changsha, China [4]. HDM-induced AR is associated with a higher risk of asthma [5, 6], the prevalence of which is increasing in many countries, especially among children. Allergen-specific immunotherapy (AIT) is the only treatment that alters the natural course of AR and prevents asthma and other allergies by inducing immunotolerance [7, 8]. Subcutaneous immunotherapy (SCIT) has been the gold standard treatment, although sublingual immunotherapy (SLIT) has emerged as an effective and safe alternative. [9, 10]. According to a recent network meta-analysis-based comparison, the symptom score-based clinical efficacy of SCIT was higher than that of SLIT drop or tablet [11]. A cluster regimen, which reduces the dose-escalation phase from 14 to 6 weeks and reduces clinical visits by 53%, is a clinical practice that facilitates the treatment of patients with tight timetables. Comparative data for cluster SCIT and conventional SCIT showed similar efficacies [12–15]. However, few studies have compared the long-term efficacy of cluster SCIT between children and adults. The present study aimed to evaluate the long-term efficacy of cluster HDM-SCIT in children and adults.

## Methods

### Patients and treatment

Patients (5–60 years old) with a clinical history of perennial HDM-induced AR for at least two years were enrolled in this study. HDM sensitisation was defined as a positive skin prick test (SPT) for *Dermatophagoides pteronyssinus* (Der p) or a specific IgE (sIgE) level against Der p ≥ 0.7 KU/L, as measured using the Pharmacia UniCAP system (Thermo Fisher Scientific China Co., Ltd., Shanghai China). Monosensitized and polysensitized patients were all eligible. Exclusion criteria included uncontrolled asthma, immunologic/systemic diseases, malignant tumours, and other conditions that were not recommended for AIT. Patients received SCIT with standardised extracts of Der p (Alutard SQ, ALK Company, Hørsholm, Denmark) at the allergy center of TongRen Hospital (Beijing, China). The dosing regimen and associated risks were determined in advance. In all the cases, it was necessary to administer a treatment course of three years, and the minimum post-treatment follow-up was three years.

### Study design

**Fig. 1:**
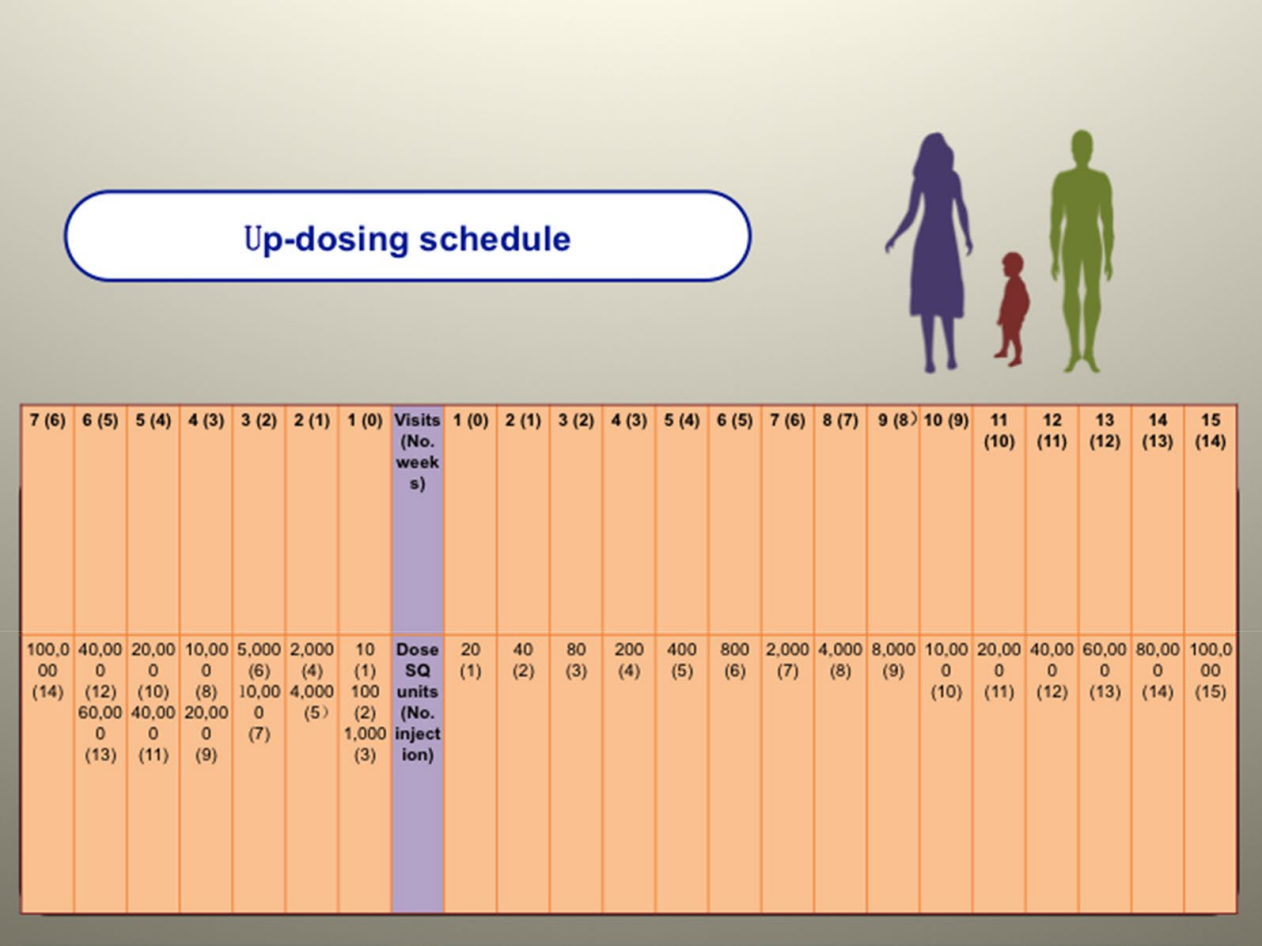
Detailed description of the updosing schedule used for cluster SCIT and conventional SCIT. Abbreviation: SCIT, subcutaneous immunotherapy; SQ, standardized extracts of Der p (Alutard SQ, ALK company, Hørsholm, Denmark)

This study was an open design, observational, long-term clinical follow-up study. To investigate the long-term efficacy of SCIT in children and adults, we continuously enrolled eligible patients for SCIT from 2005 to 2014. The build-up phase followed the cluster schedule. Elaborate regimens of the cluster versus conventional schedule are shown in Fig. 1. A total of 598 patients met the inclusion criteria and signed the informed consent forms. At baseline (T0), safety and efficacy data were collected from all eligible patients. The associated adverse events were recorded and evaluated by two nurses and one doctor in the allergy center of TongRen Hospital from baseline to the end of the three-year treatment. We obtained complete records of symptom score, medication score (MS), and mini-rhinoconjunctivitis quality of life questionnaire (RQLQmini) scores after the last injection (T1) from 490 patients (81.9%). After the last injection of SCIT, patients aged  < 18 years (n = 190) were included in the pediatric group and patients aged  > 18 years (n = 300) were included in the adult group. Post-treatment visits (T2) were scheduled for January to February 2021. Due to the unexpected coronavirus disease 2019 pandemic outbreak, an electronic questionnaire with the same contents as the paper questionnaires replaced face-to-face visits.

### Efficacy assessment

The primary efficacy endpoint was the total nasal symptom score (TNSS; maximum score = 12), the sum of four nasal symptoms (nasal blockage, runny nose, sneezing, and itchy nose), each scored from 0 (no symptoms) to 3 (severe symptoms). Key secondary efficacy endpoints included the total ocular-symptom score (TOSS), MS, combined symptom-medication score (CSMS), and RQLQmini score. TOSS (maximum score = 6) was calculated as a combination of two common ocular symptoms (gritty/red/itchy eyes and watery eyes). MS assessed the use of daily symptom-relieving medications on a four-point scale: 0, without taking medication; 1, taking antihistamines; 2, taking topical corticosteroids; and 3, taking oral corticosteroids. CSMS (score range = 0–6) equally combined the symptom scores (0–3) and medication scores (0–3). Quality-of-life assessments were based on the RQLQmini (14 questions; score rang = 0–6 for each question, maximum total score = 84). Patients who achieved TNSS improvement rates of  < 25%, 25%–65%, and  > 65% were defined as non-responders, responders, and high responders, respectively.

### Safety assessment

Nurses and doctors conducted clinical observations of adverse reactions (ADRs) for at least 30 min. The safety profile was assessed after each injection by documenting adverse events, including local ADRs (LADRs), such as wheals, redness, pruritus, and any other ADRs, and systemic ADRs (SADRs), ranging from grade 0 (no reaction or a nonspecific reaction) to grade 4 (anaphylactic shock), according to the European Academy of Allergy and Clinical Immunology (EAACI) criteria [16].

### Statistical analysis

Analyzed data showed non-normal distribution. Results are expressed as median (MED) with interquartile range (IQR). Categorical data were analysed using Chi-squared tests. Between-groups differences were analysed using Mann–Whitney U tests. Data obtained at different time points were compared using the Friedman test, and pairwise comparisons were conducted using the Wilcoxon signed-rank test with the Bonferroni correction. Correlation analysis was performed using the Pearson’s test. Statistical significance was set at p < 0.05. Statistical analyses were performed using SPSS 24.0 (IBM Corporation).

## Results

### Population characteristics

Patients (pediatric group, n = 58; adult group, n = 103; total = 161) completed the 16-year follow-up study. Monosensitization was observed at a rate of 55.18% in the pediatric group and 51.46% in the adult group. No significant differences were found in terms of sex, asthma complications, or sensitization patterns between the groups (Table 1).

**Table 1:** Demographic and survey information at baseline on the study population

Characteristic	Pediatric group (n = 58) (5–14y)	Adult group (n = 103) (15–60y)	*p* value
Age (year), median [IQR]	9 [7, 11]	31 [26, 39]	
Sex			
Male	39 (67.2%)	56 (54.4%)	*p* = 0.076
Female	19 (32.8%)	47 (45.6%)	
Asthma	7 (12.07%)	21 (20.39%)	*p* = 0.159
Sensitization pattern Monosensitization *(Der p*/*Der p* + *Der f)* Polysensitization (HDMs + ^†^others)	4 (6.90%)/28 (48.28%)	9 (8.74%)/44 (42.72%)	*p* = 0.650
	26 (44.82%)	50 (48.54%)	

Values are presented as number (%), median [IQR]

IQR, interquartile; *Der p*, *Dermatophagoides pteronyssinus*; *Der f*, *Dermatophagoides farinae*; HDMs, house dust mites

^†^Other aeroallergens beyond house dust mates

### Efficacy assessment of cluster SCIT in children and adults

**Fig. 2:**
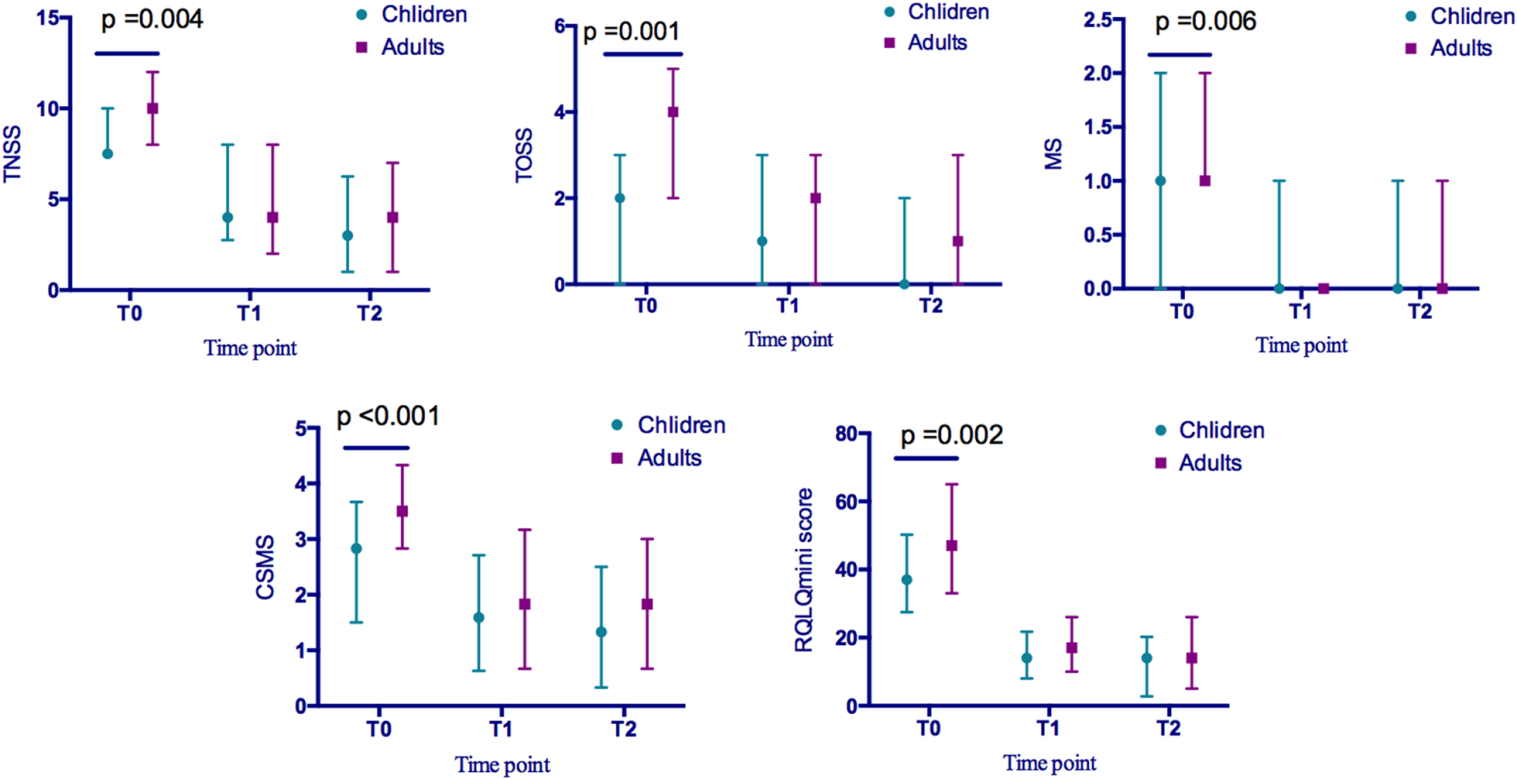
Comparison of efficacy parameters between the pediatric group and adult groups. The TNSS, TOSS, MS, CSMS, and RQLQmini scores at baseline (T0) were significantly higher in the adult group compared with those in the pediatric group. After the full course of SCIT was completed (T1) and at the end of the follow-up (T2), there was no significant difference in TNSS, TOSS, MS, CSMS, and RQLQmini scores. Abbreviations: SCIT, subcutaneous immunotherapy; TNSS, total nasal symptom score; TOSS, total ocular symptom score; MS, medication score; CSMS, combined symptom medication score; RQLQmini, mini quality of life questionnaire

**Fig. 3:**
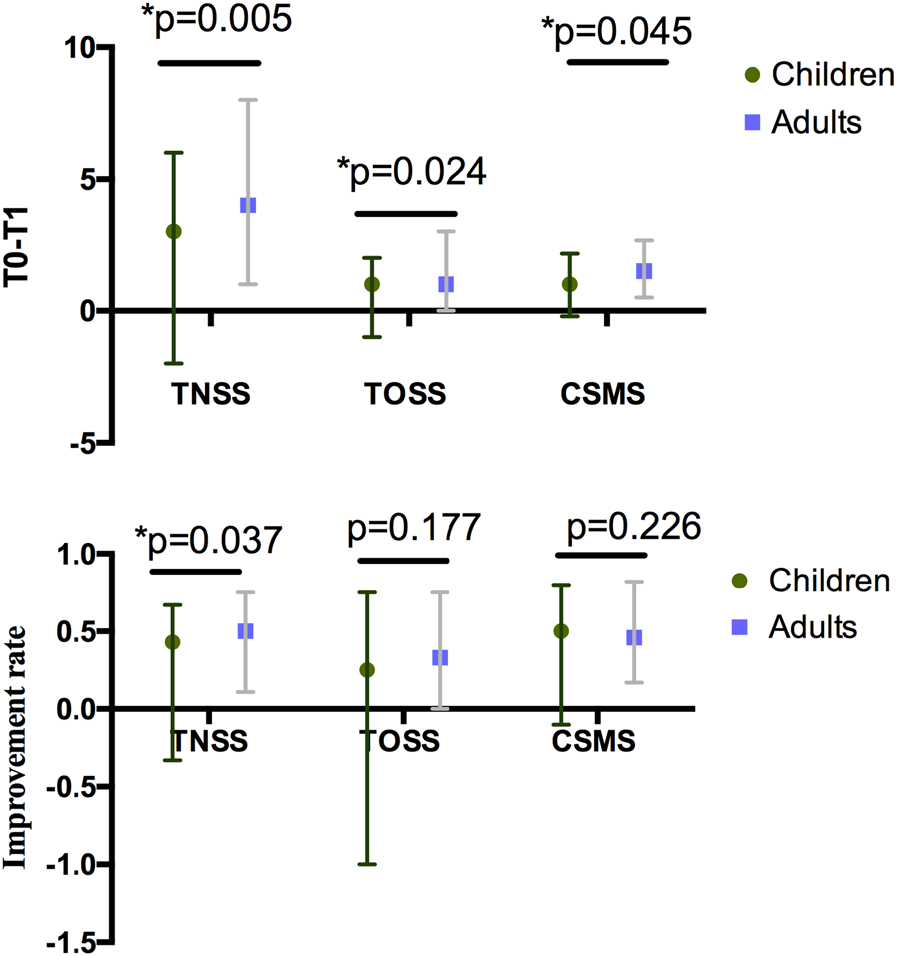
Improvements in the efficacy parameters at SCIT completion in the studied groups. TNSS, TOSS, and CSMS showed more significant improvements in the adult group than in the pediatric group. Only the improvement rate of TNSS in the adult group was significantly greater than in the pediatric group. *p < 0.05. Abbreviations: SCIT, subcutaneous immunotherapy; TNSS, total nasal symptom score; TOSS, total ocular symptom score; CSMS, combined symptom medication score

The efficacy evaluation compared the TNSS, TOSS, MS, CSMS, and RQLQmini scores in children and adults at baseline (T0) and at the end of the three-year SCIT (T1). The baseline levels of efficacy parameters in the pediatric group were all significantly lower than those in the adult group (Fig. 2). The improvement (T0-T1) in TNSS, TOSS and CSMS after SCIT was more significant in the adult group than that in the pediatric group (MED [IQR]; TNSS, 4.00 [1.00, 8.00] vs. 3.00 [− 2.00, 6.00], p = 0.005; TOSS, 1.00 [0.00, 3.00] vs. 1.00 [− 1.00, 2.00]; CSMS, 1.50 [0.50, 2.67] vs. 1.00 [− 0.21, 2.17], p = 0.024). Furthermore, the TNSS improvement rate ([T0-T1] / T0) was significantly higher in the adult group compared with that in the pediatric group (Fig. 3).

No difference was observed in the SCIT response rate between the pediatric group and adult group (nonresponders, responders and high responders; 37.93%, 27.59% and 34.48% *vs.* 30.10%, 31.07%, and 38.83%, respectively; p = 0.200). In the pediatric group, the baseline TNSS of non-responders was significantly lower than that of responders and high responders (non-responders vs. responders vs. high responders, 7 [2, 8] vs. 10 [7, 11] vs. 10 [7.5, 11.25], p < 0.01). No difference was observed in the adult subgroup comparisons (p = 0.138).

### Long-term efficacy assessments in children and adults

**Fig. 4:**
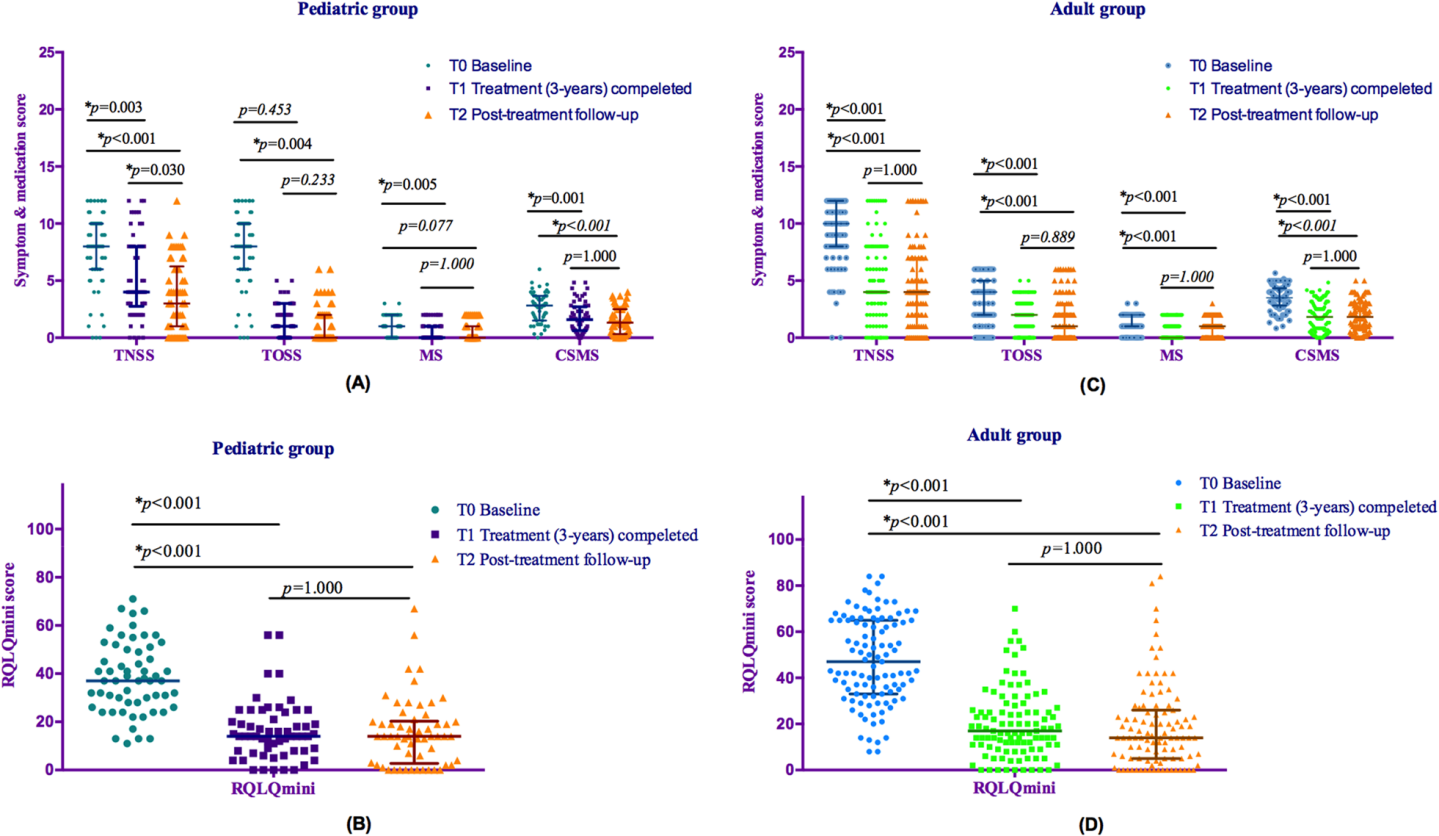
Post-treatment changes in the effectiveness of SCIT between the pediatric and adult groups. The improvements in TNSS, CSMS, and RQLQmini scores were sustained during the post-treatment follow-up (*p* < 0.01) **A**–**D**. In the pediatric group only, the TNSS decreased significantly from right after treatment to the end of the post-treatment follow-up (*p* = 0.030). *p < 0.05. Abbreviations: TNSS, total nasal symptom score; TOSS, total ocular symptom score; MS, medication score; CSMS, combined symptom medication score; RQLQmini, mini quality of life questionnaire

Withdrawal bias affected the post-SCIT follow-up duration. The minimal post-treatment follow-up duration was 3 years, and 48.45% (n = 78) patients were followed up for > 6 years after SCIT termination (MED = 5 years, IQR [4, 7.5] years). The longest post-treatment follow-up period was 13 years (n = 6, three children and three adults). The post-treatment follow-up period in the pediatric group was longer than that in the adult group (pediatric group, MED = 6 years, IQR [5, 9] years; adult group, MED = 5 years, IQR [4, 6] years; p = 0.007). In the pediatric group, until the end of the up to 13 years post-treatment follow-up (T2), TNSS, MS, CSMS, and RQLQmini scores remained significantly lower than baseline (Fig. 4A/B). TNSS was significantly lower at T2 compared with that right after SCIT cessation (T1) (Fig. 4A). In the adult group, the three-year SCIT achieved significant improvements in all efficacy indicators, TNSS, TOSS, MS, CSMS, and RQLQmini scores, and the effects of SCIT lasted until the end of follow-up (T2). No difference was observed in these parameters between T2 and T1. Details are shown in Fig. 4C/D.

In the pediatric group, 19 (86.4%) non-responders achieved extra improvement in TNSS during the post-treatment follow-up, two of whom did nor show improvements in CSMS. In adults, 39.1% of non-responders reported relatively lower TNSS at T2 than at T1, and all had improved CSMS.

### ADRs of the three-year cluster SCIT in children and adults

**Fig. 5:**
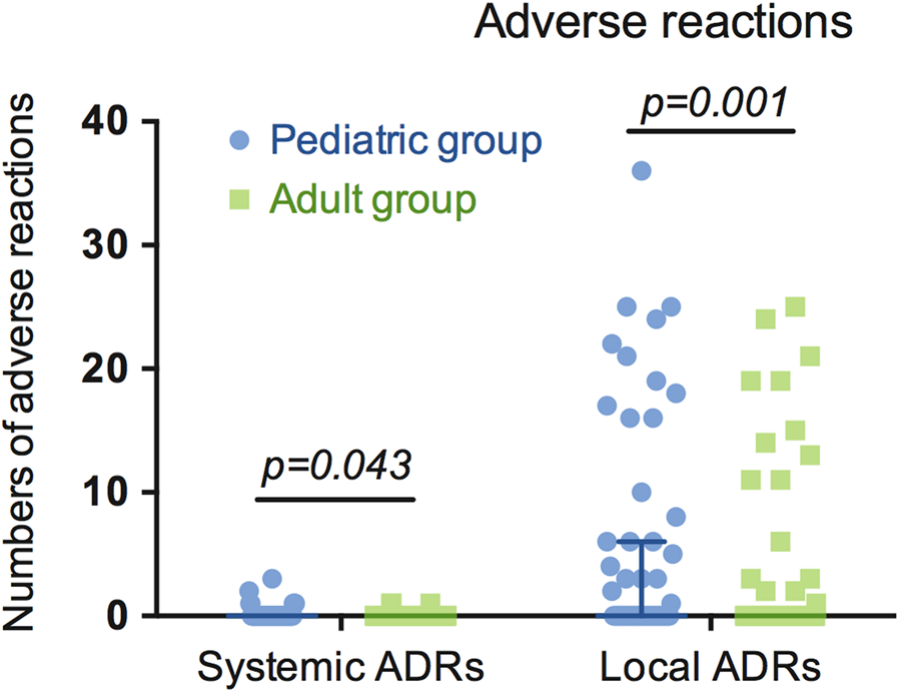
SADRs and LADRs in the pediatric and adult groups during the three-year SCIT duration. The pediatric patients experienced significantly more SADRs and LADRs than the adult patients (p = 0.043 and p = 0.001, respectively). Abbreviations: SADRs, systematic adverse reactions; LADRs, local adverse reactions

Differences in SADRs and LADRs were explored between children and adults (Fig. 5). No fatal reactions were observed. One grade 3 and six grade 1 SADRs were reported by the pediatric group, and three grade 1 SARDs were reported by the adult group (1.26% vs. 0.68% of injections, p = 0.043). The pediatric group (53.4% of injections) had more LADRs than the adult group (43.0% of injections) across the treatment duration.

### Analysis of factors correlated to the SCIT efficacy and safety

The TNSS improvement rate and long-term efficacy of SCIT (based on TNSS, CSMS and RQLQmini scores) were unrelated to SCIT safety (SADRs and LADRs). No difference in the frequency of SADRs or LADRs was observed between the monosensitization and polysensitization subgroups in the pediatric and adult groups (p = 0.236 and p = 0.479, p = 0.594 and p = 0.429, respectively).

In the pediatric group only, TNSS-based long-term efficacy was weakly negatively correlated with baseline TNSS (r = − 0.383, p = 0.003). In both groups, the TNSS (T0-T1) improvement rate was moderately correlated with the baseline TNSS (r = 0.681, p < 0.001 and r = 0.477, p < 0.001 for children and adults, respectively). The HDM-SCIT responsiveness was independent of the allergic pattern in the pediatric (p = 0.215) and adult groups (p = 0.954).

## Discussion

Although SCIT is a well-validated effective alternative for HDM-induced AR patients, it is less convenient compared with SLIT. A cluster schedule can reduce over half of the clinical visits in the updosing phase of SCIT and achieve a rapid increase in HDM-specific IgG, especially IgG4 [14], as well as an early response in clinical efficacy indicators [17]. Nevertheless, the safety risks should be taken into concern. The incidence of SADRs to SCIT was reported to vary between 0.06 and 1.01 per 100 injections [18], similar to the current study (0.68). The pediatric group showed a significantly higher risk of SADRs (1.26% per injection) compared with the adult group. However, it was lower than that reported in another study using a conventional schedule based on a pediatric population in China (4.6% per injection) [19]. LARDs are more frequent than SADRs, but are often well tolerated. Data reported by Nelson et al. showed that LADRs were experienced in 26–86% of injections [20]. The cluster schedule during the updosing phase did not influence the overall safety of the three-year SCIT compared to the previous studies using the conventional schedule.

The long-term efficacy of cluster SCIT has rarely been published, especially the direct comparison of long-term efficacy between children and adults using cluster SCIT. In this study, the minimal post-treatment follow-up duration was 3 years, and 48.45% (n = 78) patients were followed up for > 6 years after SCIT termination. The longest post-treatment follow-up period was 13 years (n = 6, three children and three adults). The TNSSs of patients who completed SCIT 13 years ago were still lower than that at baseline in the current study. A previous clinical study with a post-treatment follow-up period of 10 years (n = 20) reported no significant differences between the symptom scores obtained at three years and 10 years after HDM-specific SCIT treatment [21]. However, the sample size in this study was also limited. Another long-term follow-up study (n = 147) on patients (aged 16–25) with grass and/or birch pollen-induced AR reported improvements in rhinoconjunctivitis, and preventive effects of developing asthma of specific immunotherapy could persist for 7 years [22]. A large-scale evidence-based real-world study (n = 2350) of SCIT showed significant effects for up to 6 years (mean 3.4 years) in patients with AR, based on the number of AR medications and reductions in asthma [23]. Real-world evidence (RWE) data could reflect the efficacy of SCIT in practical applications rather than in various typical practice settings. Owing to the differences in medical service systems, reliable RWE is currently difficult to achieve in China.

To reflect the real-world application of SCIT, the severity of symptoms, use of medications, polysensitization, or a combination of asthma (controlled) were not serving as inclusion or exclusion criteria in the current study. The symptom scores, MS, and RQLQmini scores were all significantly lower in the pediatric group than those in the adult group, in line with the clinical practice. In both groups, TNSS improvements were moderately positively correlated with the baseline TNSS. This may explain the more significant TNSS improvements in the adult group compared with those in the pediatric group. Another study with a similar baseline TNSS conducted by our team revealed better improvements in children compared to adults [24]. The long-term efficacy (two-years post-SCIT) in children showed a slightly greater but not statistically significant (p = 0.905) improvement during the post-treatment years, compared to adults. However, in the current study, the improvement in TNSS post-treatment was more significant in the pediatric group than that in the adult group. To investigate the post-treatment effects of SCIT, a follow-up period > 3 years may be more valuable. The efficacy evaluation of SCIT in both the previous and current studies was based on subjective symptom scores, and the placebo effect is an issue to consider. The mean placebo effect in the SCIT trials with comparable allergen exposure (HDMs) ranged from 29.7% to 41% in the second treatment year and, in contrast, reached only 1% in the SLIT trial [25]. It has been reported that the perceived placebo effect was significantly more favorable in children than adults. [26] But in the current study, the adult group reported better TNSS improvements than the pediatric group after SCIT was completed, which the placebo effect cannot explain. O. Pfaar et al. [27] conducted a placebo-controlled study with an HDM allergoid SCIT in allergic rhinoconjunctivitis patients. They reported that after the first 6 months of treatment, a similar improvement was observed in both the treatment groups and in the placebo group, but at 12 months, a further decrease in the treatment group was observed while the decrease in the placebo group remained approximately the same. The same phenomenon was seen in several AIT studies [28, 29]. That means the placebo effect does have a significant limitation compared with the treatment effect induced by SCIT. In addition, longer follow-up duration was associated with a smaller placebo effect size [30]. The additional improvement in TNSS, after SCIT cessation, in the pediatric group may have no relationship with the placebo effect. A 10-year follow-up cohort study showed persistent improvements in rhinoconjunctivitis and the potential to prevent asthma development in children with AR for up to seven years. [31] The effect of slowing asthma progression is more pronounced in children than in adults [32]. The different long-term benefits suggest that the influence of immunotherapy on allergic symptoms may vary in adults and children. The HDM-SCIT responsiveness was independent of the allergic pattern in the pediatric and adult groups in the current study. The result was consistent with the previous findings of Song et al. who found that single-allergen SCIT is beneficial for treating AR caused by multiple allergens in pediatric populations [33].

This is the first study to report that TNSS may continuously improve beyond immunotherapy termination in children who complete a three-year SCIT during childhood. The baseline TNSS in the pediatric group was negatively correlated with post-treatment benefits. Furthermore, 86.4% of non-responders in the pediatric group showed further improvement in TNSS during the post-treatment follow-up. Regarding the influence of pharmacotherapy, CSMS improved in 17(77.3%) pediatric AR patients after the termination of SCIT. In our previous study, patients with a history of AR < 10 years maintained better HDM-SCIT efficacy during post-treatment (two-year) observation [24]. Children who completed SCIT during childhood with a low baseline TNSS may gain additional benefits later. The mechanisms of AIT are still not fully understood. Tolerance is accompanied by Th1/Th2 rebalancing, changes in secretory cytokines, production of IgG4 isotype allergen-specific blocking antibodies, induction of regulatory subsets of T and B cells (Tregs and Bregs), and a decrease in inflammatory responses to allergens by effector cells (mast cells, basophils, and eosinophils) and upstream dendritic cells (DCs) in inflamed tissues [33]. The reported mechanism studies of AIT were mostly based on adult populations. The immune systems of children are not fully matured. The impact of immunotherapy on allergic diseases in children may more profound. The mechanism of the long-term efficacy achieved with AIT in children merits further study.

The current study has some limitations. Post-treatment dropouts were relatively high. The limited sample size did not enable further stratified analysis of patients with different post-treatment follow-up durations. As blood samples were not collected, evaluation of biomarkers in patients with different responsiveness and long-term outcomes was not possible.

## Conclusions

Children and adults with HDM-induced perennial AR achieved sustainable efficacy of a three-year SCIT for at least 3 years (up to 13 years) after treatment. Patients with relatively severe nasal symptoms may show more significant improvement after treatment. Children who complete a full course of SCIT in childhood may gain further improvement after SCIT cessation, regardless of the responses immediately after the immunotherapy.

## Data Availability

The datasets used and/or analysed during the current study are available from the corresponding author on reasonable request.

## Abbreviations

AR: Allergic rhinitis
HDMs: House dust mites
AIT: Immunotherapy
SCIT: Subcutaneous immunotherapy
SLIT: Sublingual immunotherapy
SPT: Skin prick test
*Der p*: *Dermatophagoides pteronyssinus*
sIgE: Specific IgE
TNSS: Total nasal-symptom score
TOSS: Total ocular symptom score
MS: Medication score
CSMS: Combined symptom medication score
RQLQmini: Mini-rhninoconjunctivetis quality-of-life questionnaire
EAACI: European academy of allergy and clinical immunology
ADRs: Adverse reactions
LADRs: Local adverse reactions
SADRs: Systematic adverse reactions
IQR: Interquartile
Tregs: T-regulatory cells
Bregs: B-regulatory cells
DCs: Dendritic cells
